# Development of summer skiing days in Austrian glacier ski areas in the first two decades of the twenty-first century

**DOI:** 10.1007/s00484-022-02371-6

**Published:** 2022-10-01

**Authors:** Marius Mayer, Bruno Abegg

**Affiliations:** 1 Department of Strategic Management, Marketing and Tourism, SME & Tourism, Faculty of Business and Management, University of Innsbruck, Universitätsstraße 15, 6020 Innsbruck, Austria; 2 Faculty of Tourism, Munich University of Applied Sciences, Schachenmeierstraße 35, 80636 München, Germany; 3 Institute for Systemic Management and Public Governance, University of St. Gallen, Dufourstrasse 40a, 9000 St. Gallen, Switzerland

**Keywords:** Glacier ski areas, Climate change, Summer skiing, Decline, Operating days, Austria

## Abstract

Summer skiing on glaciers is a leisure activity highly dependent on natural factors like weather and glacier conditions. While the literature agrees that climate change is shaping the fate of summer skiing, longer time series covering both the supply and demand side of glacier/summer skiing, and research that combines natural and non-natural factors affecting the summer ski market are missing. To close this research gap, we conducted a detailed supply-side analysis of nine Austrian glacier ski areas focusing on the number of operating days (in the summer half-year, as well as for meteorological and astronomical summer ski definitions), show how these numbers evolve over time, test how they correlate with meteorological and glaciological data using time-series regression models on a yearly and monthly basis, and highlight how managerial decisions come into play when trying to explain the summer ski supply’s decline. Between 2002 and 2019, summer ski operating days in Austrian glacier ski areas declined by 48.3% in the summer half-year, 65.2% in the meteorological, and 62.3% in the astronomical summer parallel to rising mean temperatures and shrinking glaciers. This decrease is strongest in June to September and weakest in May and October but in two glacier ski areas, the operating days in the summer season remain constant or are even on the rise. This is in line with model results indicating that meteorological and glaciological data only explain parts of the variance of the decline trends. Operators’ agency, strategies, and decisions play an important role underlining that global warming is not monocausally determining summer ski operation.

**Supplementary Information:**

The online version contains supplementary material available at 10.1007/s00484-022-02371-6.

## Introduction

Global warming is rapidly changing the cryosphere in the world’s high mountain areas, including Europe (Hock et al. 2019) and Austria (Fischer et al. 2015). From 1969 to 2006, the Austrian glaciers lost 26% of their area and 30% of their volume (Helfricht et al. 2019). For the subsequent decade up to 2016, an additional ice volume loss of 22% - pointing to an accelerated glacier retreat (Sommer et al. 2020) - was calculated (Helfricht et al. 2019). Glacier loss is seriously affecting water resources, disaster risk management, hydropower generation, agriculture, and tourism (Hock et al. 2019). Different types of glacier tourism are known including sightseeing, trekking/mountaineering, educational tours, and glacier skiing (Welling et al. 2015; Salim and Ravanel 2020; Salim et al. 2021a, b). Basically, glacier skiing can be practiced year-round on the glaciers’ accumulation areas - in this contribution, however, we focus on glacier skiing in the summer half-year as being expected to be most vulnerable to climate change impacts. However, a clear, commonly accepted definition of summer skiing does not exist in the literature as highlighted by Hupke (1990) and König (1994). While a broad perspective (e.g., Barnick 1970) sees summer ski areas as being in operation between May and November (i.e., the summer half-year, SHY), narrower perspectives focus on the meteorological (MET, June 1 to August 31) and the astronomical summer (ASTR, June 21 to September 21) definitions (e.g., Mayer 2012). Given the variations in temperature, snowpack, insolation, and, as a consequence, glacier status over the SHY, it is obvious that these differing understandings of summer skiing have enormous consequences for the amount of operating days in the respective periods and, especially in the light of ongoing climate change, on their future development. The broad perspective on the SHY overshadows the particularities of the actual summer season by intermingling with the spring and autumn seasons which are characterized by very different natural and demand conditions. The missing clear definition also leads to confusion as exemplified for Stubaier Glacier, Austria: While Lemper and Schröder (2011) as well as Carver and Tweed (2021) report the last year of summer ski operation to be 1998, the operators themselves speak of 2002 (pers. comm.) - both without referring to any definition - while our data reveal MET summer ski operation for all years until 2019 except for 2016 respectively ASTR summer ski operation for each year except for 2012, 2016, and 2018 (see Supplements 2b and c). Obviously, summer skiing is mixed-up with year-around skiing or skiing throughout high summer. This ambiguity also influences research results. Falk (2016), looking at the SHY transportation frequencies of Austrian glacier ski areas (GSAs) between 1972 and 2007, could not find any evidence of a climate change-induced decline as his data set also includes May and October - two shoulder season months which are much less affected by climate change impacts on the glaciers compared to the MET and ASTR summer seasons but which show relatively high frequentation in the GSAs (Haimayer 1989; Mayer et al. 2018). The SHY definition of summer skiing is also at odds with the operators’ perspective on summer skiing which is clearly focused on the actual summer months: June to September (Stubaier Glacier) or July to September (Kitzsteinhorn) (pers. comm.).

Regarding the significance of the summer ski season for the Austrian GSAs in comparison to the winter season, there are no data available for all GSAs. Traditionally, the most important months for the Austrian GSAs were (and most likely still are) October and November as well as March and April (Haimayer 1989). In Hintertux, the only remaining all-year around GSA in Austria, circa 2000 first entries were recorded on summer days with good weather conditions (data from 2009); among them are circa 400 leisure skiers, 600 athlete skiers, and 1000 excursionists (Ehrne and Hilpert 2011). For the Kitzsteinhorn, the first entries between November 2005 and May 2013 indicate a winter share of 78.9% (November to April) vs. a summer share of 21.1% (May to October) (Mayer et al. 2018). These first entry data include excursionists, which is also the case for the official Austrian ropeway and ski-lift frequentation statistics (stopped after 2007). According to this database, the share of GSAs among the total number of passengers transported uphill in the summer half year accounts for less than 1% (Falk 2016). These information underline that summer skiing is a niche market for the Austrian GSAs with a limited economic relevance. Thus, the relevance of summer skiing is not due to its volume but rather based on its symbolic dimension, traditionally signifying snow reliability all-year around and providing access to spectacular high-alpine sceneries also for non-skiers. In addition, most Austrian GSAs are the smaller parts of much larger ski areas in the winter season (see Supplement 1).

Direct climate change impacts on summer skiing include retreating glaciers and therefore smaller areas available for skiing, less frequent summer snowfall, and icy slopes (Fischer et al. 2011; Steiger et al. 2021). Given the close links between this specific activity and its physical base, it is not surprising that summer skiing was discussed as the “first victim of climate change” (Mayer 2012), as an example of “last chance tourism” (Steiger et al. 2012), and as a tourism activity “on the brink of collapse” (Schmude and Berghammer 2015).

Most summer ski areas were established in the cooler and more “glacier-friendly” 1960s and 1970s (Fischer et al. 2011). Demand for summer skiing peaked in the 1980s (Haimayer 1989). Shortly after, and coinciding with the beginning of the accelerated warming trend (Olefs et al. 2021) and the resulting negative mass balances of the glaciers (Helfricht et al. 2019), demand started to decline. König (1994), investigating the GSAs in Switzerland, was likely the first researcher to call summer skiing a shriveling market. Diolaiuti et al. (2006) confirmed this general trend. However, they also found that favorable climatic conditions in a single year can result in more glacier surface area being available for skiing, better skiing conditions, and, consequently, higher visitor numbers. This interrupts - at least temporarily - the prevalent declining trend in summer skiing. Falk (2016), surprisingly, did not find a shriveling but a stagnating summer ski market in Austria. A closer look at the data reveals that his data also includes May and October and that he did not distinguish between skiing and non-skiing visitors (note that the latter usually account for the majority of summer visitors, Haimayer 1989; for Kitzsteinhorn, Mayer et al. (2018) reported an estimated share of non-skiers/excursionists during the summer half year of 57% for the 2005–2013 period). Mayer (2012) focused on the number of operating GSAs. He used daily data (i.e., GSA in operation yes/no), covering the entire Alps but only 1 year (2011), and showed that the number of operating GSAs greatly varied across the year. More recently, Mayer et al. (2018) conducted a detailed demand analysis for one Austrian GSA covering the time period from 11/2005 to 04/2013. Weather data but no glaciological parameters were used to explore the volatility and sensitivity of different demand indicators. They found a significant volatility in ski area visitation, especially during the summer seasons, and that snow depth has no significant influence on summer skiing frequentation, which, at first glance, contradicts the presumed negative effects of climate change. Demiroglu et al. (2018) investigated summer skiers’ perception of and adaptation to climate change in Norway, while Carver and Tweed (2021), showcasing the Stubai GSA (Austria), focused on the operators’ strategies to cope with deglaciation. Regarding technical adaptation measures to reduce snow and ice ablation (e.g., Fischer et al. 2016; Senese et al. 2020; Huss et al. 2021), the covering of glaciers with geotextiles was found to be the most effective measure (e.g., ablation on Swiss test sites was reduced by 60%). Its application, however, is costly, limited to relatively small areas and “can only delay the decay of glaciers in ski resorts” (Fischer et al. 2016, p. 2950).

The cited studies pointed to the importance of climate change in shaping the fate of summer skiing. A few scholars, however, stated that the decline in summer skiing cannot be explained by rising temperatures and shrinking glaciers alone (Abegg et al. 1994; Mayer 2012; Steiger et al. 2021). König (1994), for example, noted that summer skiing lost its original appeal as an exclusive activity. Competition arose from new outdoor activities, and from the ever better established mid- and long-haul (beach) destinations. Mayer (2012) found a “vicious circle,” where physical (i.e., the impacts of climate change) and operational challenges (i.e., rising costs) led to a steady reduction in summer ski supply which, in turn, further affected an already declining demand (due to a negative image and low price-performance-ratios). The existing literature provides some interesting, albeit highly fragmentary insights. What is missing, for example, are longer time series covering both the supply and demand side of glacier/summer skiing, and research that combines the natural (e.g., glaciological and meteorological) and non-natural factors (e.g., managerial decisions) affecting the summer ski market. This paper aims to address some of these knowledge gaps by conducting a detailed analysis of all nine Austrian GSAs. We focus on the number of their operating days (summer half-year, meteorological, and astronomical summer ski definition), show how these numbers evolve over time (2002 to 2019), test how they correlate with meteorological and glaciological data, and highlight how managerial decisions come into play when trying to explain summer ski’s decline.

## Materials and methods

In Austria, nine GSAs opened between 1966 and 1987. They vary considerably in size, altitudinal range, and importance for the overall ski area (Supplement 1). Historically, all GSAs offered summer skiing but since the early 1980s they are affected by climate change-induced glacier shrinkage (Fischer et al. 2016) leading to a loss of skiable surface, steeper slopes (more challenging for beginners and less suitable for large crowds), dis- or replacement of lifts, and continuing adaptation efforts of the operators.

For the analysis of the GSAs’ development, both demand and supply-side data would be important. However, as detailed skier day numbers are not available for most GSAs, we concentrate on the supply-side,^1^ which is potentially crucial due to the operators’ agency. Thus, we analyze the development of operating days for the three definitions of summer ski season explained above: summer half year (SHY, May 1 to October 31), the meteorological (MET, June 1 to August 31), and the astronomical summer (ASTR, June 21 to September 21) for the 2002 to 2019 period. We deliberately opted to analyze the operating days for these three differing summer ski season definitions because there is no generally accepted summer ski definition and the preference of one of them would be arbitrary. The analysis of these different time periods should control whether the results vary depending on the differing understanding of the summer ski season. We assume the results to vary since May and October, the two busiest months of the summer half-year in terms of skier frequentation in the GSAs, are not included in the MET and ASTR summer ski season definitions focusing on the actual summer months. MET and ASTR summer ski seasons are also assumed to vary due to the glaciological differences between June (usually plenty of winter snow) and September (bare ice prevalent) which should impact summer ski operations. The year 2002 emerged as a suitable starting point as detailed operating data is available from this year on. This also coincides with the beginning of the decrease in summer ski operation (in the 1990s, ski operation all summer long was still common in Austrian GSAs, see Hutter 2005). 2019 is the last summer to be considered before the Covid-19 pandemic overruled all other factors affecting ski tourism in Austria (Mayer et al. 2021). We compiled the daily operating status from various sources: operators’ internal data, user reports and postings in skiers’ online communities (sommerschi.com and alpinforum.com), newspaper articles (accessed via the Austrian Press Agency), and website/webcam archives like wayback machine. We aggregated the summer ski operating days and calculated their share in relation to potential maximal operating days.^2^

As detailed meteorological and glaciological data is not available for the GSAs, we use proxy variables representative for the higher altitudes of the Austrian Alps (as asserted by experts from the Austrian Central Institution for Meteorology and Geodynamics^3^): Weather data (seasonal, monthly and daily means of temperature, seasonal sunshine hours, monthly and daily snow depth values^4^) is taken from Hoher Sonnblick observatory, 3106 m asl in the Hohe Tauern region, eastern central Alps (ZAMG 2021), as well as from the Vernagtferner meteorological station (2640 m asl) in the Ötztaler Alps, Tyrol in Western Austria (Bayerische Akademie der Wissenschaften 2021a). Glacier mass balances, the accumulation area ratio^5^ (AAR), and the equilibrium line altitude^6^ (ELA) come from two long-term time series: Vernagtferner (Bayerische Akademie der Wissenschaften 2021b) and Stubacher Sonnblickkees (Slupetzky and Ehgartner 2014; Zagel et al. 2021), representing Western and Eastern Austrian glaciers. Regarding the influences of meteorological and glaciological variables we expect the following relationships (Table 1).

**Table 1:** Expected relationships between summer ski operating days and meteorological and glaciological variables

Variable	Expected Sign	Explanation/Assumptions
Temperature (SHY, summer)	Negative	The higher the temperature, the lower the number of operating days. Higher temperatures increase snow and ice melt which worsens skiing conditions
Snow depth	Positive	The larger the snow depth, the higher the number of operating days. Higher amounts of snow take more time to melt and indicate better skiing conditions
Sunshine duration	Negative	The higher the sunshine duration, the lower the number of operating days. Longer sunshine duration corresponds to higher temperatures, increased snow/ice melt and worse skiing conditions
Glacier mass balance (MB)	Positive	The more positive the MB, the higher the number of operating days. A positive MB stands for relatively cool summer temperatures and snowfall all year around. This corresponds to better skiing conditions
Equilibrium line altitude (ELA)	Negative	The higher the ELA, the lower the number of operating days. A high ELA means that large parts of the glacier’s altitudinal range show a net ice mass loss as the complete winter snow is melted and bare ice is exposed. This corresponds to unsuitable skiing conditions
Accumulation area ratio (AAR)	Positive	The higher the AAR, the higher the number of operating days. Glacier areas are part of the accumulation area, if they show a net gain of snow after a summer season and do not expose bare ice. The accumulation area thus provides suitable skiing conditions

These meteorological and glaciological data were correlated with the share of summer ski operating days using Pearson correlations; in order to check whether there are general influences of meteorological and glaciological trends on the summer ski operating days, we start these correlation analyses with the aggregated shares of operating days (Table 2) and present the correlations with the separate GSAs in the supplementary data (Suppl. 4).

**Table 2:** Correlations of summer ski operating days in Austrian GSAs with (a) meteorological and (b) glaciological data, aggregated level

Pearson R	Operating days aggregated withoutHintertux		
	Summer half-year	MET summer ski	ASTR summer ski
a) Meteorological variables			
Vernagtferner Temperature Summer (Jun-Sept)	− 0.556*	− **0.405**^**#**^	− **0.469**^**#**^
Hoher Sonnblick Temperature Summer (Jun–Aug)	− 0.583*	− 0.574*	− 0.630**
Hoher Sonnblick Temperature SHY (April–Sept)	− 0.744***	− 0.663**	− 0.708**
Hoher Sonnblick Sunshine duration Summer (Jun–Aug)	− 0.201	− 0.227	− 0.273
Vernagtferner av. snow depth (May 1) converted to water equivalent mm	− 0.211	− 0.116	− 0.124
Hoher Sonnblick av. snow depth (May 1)	− 0.098	− 0.115	− 0.065
Hoher Sonnblick mean snow depth SHY	− 0.033	− 0.100	0.014
Hoher Sonnblick mean snow depth MET summer	0.004	− 0.045	0.044
Hoher Sonnblick mean snow depth ASTR summer	0.033	− 0.021	0.099
b) Glaciological variables			
Vernagtferner mass balance	**0.436**^**#**^	0.379	0.489*
Stubacher Sonnblickkees mass balance	0.195	0.211	0.299
Vernagtferner ELA	− 0.272	− 0.207	− 0.322
Stubacher Sonnblickkees ELA	− **0.452**^**#**^	− **0.448**^**#**^	− 0.533*
Vernagtferner AAR	0.507*	**0.447**^**#**^	0.525*
Stubacher Sonnblickkees AAR	0.383	0.371	**0.441**^**#**^

Statistically significant correlation results are highlighted in bold

^***^ *p* < 0.001, ^**^p < 0.01, ^*^p < 0.05, ^#^*p* < 0.1

Considering the data structure with time series observations of eight different GSAs,^7^ we used panel and time series regression models based on regular OLS estimators (see Giesselmann and Windzio 2012) to explain the variance of the summer ski operating days from different time perspectives: We analyzed the yearly operating day trends (*n* = 144), with the seasonal variations SHY, MET summer ski, and ASTR summer ski, given that glacier data is also reported on a half-year basis, using panel regressions to observe many destinations over a period of time (see Table 3) as well as time series regressions, where we observe one destination over a period of time (see Table 4). Second, to better control for the seasonal variation, we also ran monthly panel regression models (*n* = 864) (see Table 5). These models were built up step-wise. That means we first tested for a time trend (using years), then added meteorological and/or glaciological variables, their combinations, dummy variables for the separate GSAs as fixed effect variables, and monthly dummies. In the monthly models, monthly snow average values replace the glacier data not available on a monthly basis. The models were characterized by high autocorrelation (typical for time series data). Therefore, we used the Cochran-Orchutt and the Prais-Winsten procedures in SPSS20® to correct for autocorrelation (Prais and Winsten 1954; Demiroglu et al. 2015), with the final results being taken from the Prais-Winsten models due to higher explanatory power and Durbin-Watson autocorrelation measures closer to their optimal value of two (Dielman 1985). Models were selected based on their explanatory power (adjusted R^2^), the absence of too high autocorrelation, and the maximum of statistically significant independent variables.

**Table 3:** Panel regression models using the Prais-Winsten-procedure based on yearly data (*n* = 144)

	R^2^ adj	Year	Standardized Beta Values		
			Temperature SHY	Glacier (ELA Vernagt)	GSA Fixed Effect
a) SHY					
Model 1	0.195	− 0.454***			No
Model 2	0.309	− 0.350***	− 0.357***		
Model 3	0.331	− 0.343***		− 0.388***	
Model 4	0.345	− 0.323***	− 0.184^#^	− 0.269**	
Model 5	0.495	− 0.439***			Yes
Model 6	0.527	− 0.344***	− 0.276***		
Model 7	0.516	− 0.401***		− 0.283***	
Model 8	0.525	− 0.361***	− 0.159*	− 0.187*	
b) MET					
Model 1	0.172	− 0.429***			No
Model 2	0.241	− 0.344***	− 0.282*** (SUM)		
Model 3	0.273	− 0.329***		− 0.338***	
Model 4	0.270	− 0.324***	− 0.067 (SUM)	− 0.287*	
Model 5	0.490	− 0.392***			Yes
Model 6	0.499	− 0.330***	− 0.190*** (SHY)		
Model 7	0.493	− 0.339***		0.199** (AAR Vernagt)	
Model 8	0.490	− 0.344***	− 0.056 (SHY)	0.189* (MB Vernagt)	
c) ASTR					
Model 1	0.133	− 0.380***			No
Model 2	0.274	− 0.261**	− 0.398*** (SUM)		
Model 3	0.350	− 0.235**		− 0.490***	
Model 4	0.348	− 0.229**	− 0.075 (SUM)	− 0.433***	
Model 5	0.383	− 0.353***			Yes
Model 6	0.408	− 0.263***	− 0.276*** (SHY)		
Model 7	0.412	− 0.288***		0.375*** (MB Vernagt)	
Model 8	0.410	− 0.275***	− 0.081 (SUM)	0.312** (MB Vernagt)	

***p < 0.001, **p < 0.01; *p < 0.05; ^#^*p* < 0.1

Temperature: SHY Hoher Sonnblick mean summer half-year April to September; SUM: Hoher Sonnblick mean summer June to August

**Table 4:** Time series regression models using the Prais-Winsten-procedure based on yearly data (*n* = 18)

	Stubai	Kaunertal	Kitzsteinhorn	Rettenbachferner	Tiefenbachferner	Pitztal	Mölltal	Dachstein
a) SHY								
R^2^ adj	0.618	0.549	0.465	0.080	0.205^a^	0.436	− 0.008	0.431
Year sig	− 0.814***	− 0.776***	− 0.726**	− 0.434^#^	− 0.547*	− 0.709**	0.333	− 0.706**
R^2^ adj	0.688	0.570	0.459	0.418	0.148	0.462	− 0.070	0.623
Year	− 0.539**	− 0.666**	− 0.564*	− 0.173	− 0.529*	− 0.651**	0.370	− 0.511**
Temp (SHY)	− 0.452*	− 0.308^#^	− 0.328	− 0.596*	− 0.055	− 0.180	− 0.063	− 0.471*
R^2^ adj	0.791	0.520	0.583	0.384	0.193	0.397	0.021	0.615
Year	− 0.744***	− 0.744**	− 0.637**	− 0.233	− 0.510*	− 0.697**	0.338	-0.666**
Glacier	0.373** AAR Vernagt	0.160 MB Vernagt	0.523** MB Sonnblickkees	0.637** MB Vernagt	0.200 AAR Vernagt	0.073 MB Vernagt	− 0.256 ELA Vernagt	− 0.484** ELA Vernagt
b) MET								
R^2^ adj	0.585^b^	0.542	0.308	0.005	0.160^a^	− 0.002	0.325	0.493
Year	− 0.780***	− 0.762***	− 0.624**	− 0.349	− 0.508*	− 0.341	0.636**	− 0.744**
R^2^ adj	0.656	0.492	0.538	0.194	0.225^a^	0.004	0.383	0.659
Year	− 0.578**	− 0.758**	− 0.331^#^	− 0.194	− 0.598*	− 0.257	0.783**	− 0.619**
Temp	− 0.403* SHY	− 0.020 SHY	− 0.642** SUM	− 0.501* SUM	0.308 SUM	− 0.243 SUM	− 0.332 SUM	− 0.400* SHY
R^2^ adj	0.745	0.494	0.538^c^	0.214	0.134^a^	− 0.059	0.314	0.659
Year	− 0.728***	− 0.767**	− 0.504**	− 0.218	− 0.528*	− 0.353	0.611**	− 0.707***
Glacier	0.364* AAR Vernagt	− 0.063 MB Vernagt	0.620** MB Sonnblickkees	− 0.527* ELA Sonnblickkees	0.163 ELA Sonnblickkees	− 0.132 MB Vernagt	0.195 MB Sonnblickkees	− 0.441** ELA Vernagt
R^2^ adj	No models with sig. year, temp and glacier variables		0.613^a^	No models with sig. year, temp and glacier variables				
Year			− 0.350*					
Temp			− 0.407^#^					
Glacier			0.400^#^ MB Sonnblickkees					
c) ASTR								
R^2^ adj	0.504^b^	0.262	0.434	0.055	0.160^a^	− 0.021	0.175	0.669
Year	− 0.730**	− 0.591*	− 0.708**	− 0.408	− 0.508*	− 0.315	0.521*	− 0.842***
R^2^ adj	0.566	0.263	0.623	0.319	0.227^a^	0.057^b^	0.312	0.818
Year	− 0.483*	− 0.521*	− 0.416*	− 0.156	− 0.598*	− 0.170	0.686**	− 0.649***
Temp	− 0.464* SHY	− 0.278 SHY	− 0.624** SUM	− 0.595* SHY	0.310 SUM	− 0.300 SHY	− 0.416^#^ SUM	− 0.414** SHY
R^2^ adj	0.619	0.261	0.637	0.357	0.135^a^	0.217^b^	0.187	0.807
Year	− 0.445*	− 0.546*	− 0.530**	− 0.206	− 0.528*	− 0.349	0.565*	− 0.494***
Glacier	− 0.671** ELA Vernagt	0.239 AAR Vernagt	0.662*** MB Sonnblickkees	− 0.640** ELA Vernagt	− 0.528 ELA Sonnblickkees	0.459^#^ MB Sonnblickkees	− 0.255 ELA Sonnblickkees	− 0.732*** ELA Vernagt
R^2^ adj	No models with sig. year, temp and glacier variables		0.715	No models with sig. year, temp and glacier variables	0.756	No models with sig. year, temp and glacier variables	0.365	0.812
Year			− 0.378*		− 0.828***		0.606*	− 0.483***
Temp			− 0.404*		0.970*** SUM		− 0.751* SUM	− 0.163 SHY
Glacier			0.444* MB Sonnblickkees		0.545** AAR Vernagt		− 0.502* AAR Vernagt	− 0.619** ELA Vernagt

***p < 0.001, **p < 0.01; *p < 0.05; ^#^*p* < 0.1﻿; standardized beta values in lines 3, 5–6, 8–9; 11–13; Temperature: SHY Hoher Sonnblick mean summer half-year April to September; SUM: Hoher Sonnblick mean summer June to August

^a^Durbin-Watson value 1.361

^b^Without Praise-Winsten-procedure

^c^Durbin-Watson value 1.340.

**Table 5:** Panel regression models using the Prais-Winsten-procedure based on monthly data (*n* = 864) covering SHY

	Model 1	Model 2	Model 3	Model 4	Model 5	Model 6	Model 7	Model 8	Model 9	Model 10	Model 11
R^2^ adj	0.020	0.157	0.032	0.179	0.104	0.266	0.265	0.390	0.398	0.440	0.440
Year	− 0.150***	− 0.136***	− 0.138***	− 0.120***	− 0.175***	− 0.163***	− 0.149***		− 0.118***	− 0.151***	− 0.155***
Temp. Sonnblick		− 0.369***		− 0.381***		− 0.378***	− 0.387***		− 0.062	− 0.065	
Snow Sonnblick			− 0.122***	− 0.154***			− 0.116***		− 0.003	0.005	
GSA Fixed Effect	No	No	No	No	Yes	Yes	Yes	No	No	Yes	Yes
June								− 0.205***	− 0.159**	− 0.146**	− 0.191***
July								− 0.237***	− 0.174*	− 0.156*	− 0.222***
August								− 0.477***	− 0.414***	− 0.379***	− 0.445***
September								− 0.294***	− 0.269**	− 0.245**	− 0.276***
October								0.387***	0.376***	0.360***	0.358***

***p < 0.001, **p < 0.01; *p < 0.05; ^#^*p* < 0.1

Standardized beta values in lines 3 and below; Temp.: mean monthly temperature Hoher Sonnblick observatory (3106 m asl); Snow: monthly mean snow values of Hoher Sonnblick observatory 3106 m asl calculated based on daily average values; Reference category for GSA fixed effect: Mölltal; Months dummies: May

Background information about operators’ strategies come from eight personal interviews (average length of 78.6 min, fully transcribed and analyzed using qualitative content analysis) and were complemented by email exchanges, own observations, skiers’ online communities, press releases and newspaper articles.

## Results

### Development of summer ski operating days in Austrian GSAs

**Fig. 1:**
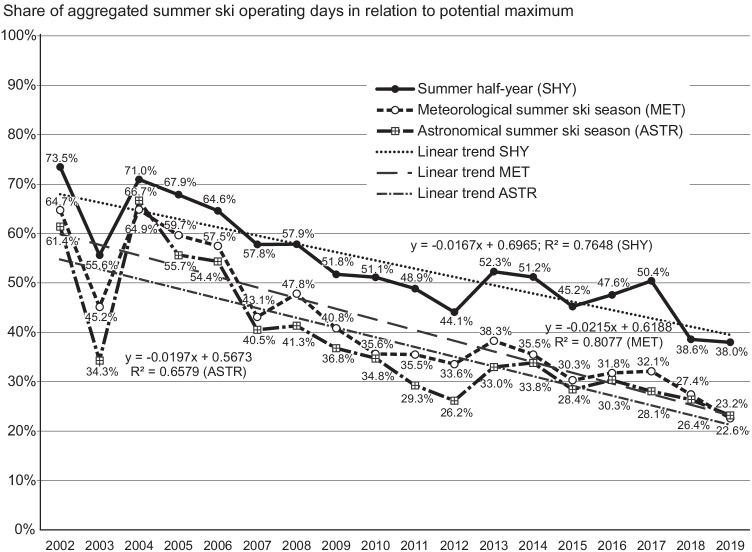
Development of summer ski operating days in Austrian glacier ski areas 2002–2019 (aggregated including Hintertux)

In 2002, the Austrian GSAs offered 1217 SHY ski days out of a potential maximum of 1656 (73.5%), followed by a pronounced decline (Fig. 1). Even though all GSAs still offer skiing in the SHY, the operating level declined by 48.3% to 38.0% of the potential operating days (2019). MET summer ski was offered in 2002 by eight of nine GSAs with 64.7% of the potential operating days. As for 2019, only four GSAs offer MET summer ski adding up to only 22.6% (− 65.2%) of the potential operating days. ASTR summer ski offer reduced from nine GSAs (61.4%) in 2002 to six GSAs (23.2%), which equals a decline by 62.3%.

At the destination level, the development of the summer ski operating days shows a large variation (Supplement 2a-c): In the SHY, all GSAs except Hintertux show a decline in operating days, however, varying between 9.4% for Mölltal and 98.7% for Dachstein (Supplement 2a).

The MET summer ski development on the destination level shows a clearer, more diverging picture (Supplement 2b): While Hintertux remained constant and Mölltal increased its operating days (15 days, 33.3% between 2002 and 2019), Pitztal offered no MET summer ski (with two exceptions), Tiefenbach- and Rettenbachferner stopped MET summer ski operations completely after 2003 and 2006, Kitzsteinhorn reduced operating days in the MET summer considerably after 2007/08 as did Kaunertal and Dachstein to partly reach the zero level, and Stubai showed a strong decline with positive outliers.

Regarding ASTR summer ski (Supplement 2c), the special case of Mölltal is even more pronounced with an increase of 84.4% between 2002 and 2019 (from 32 to 59 days). Tiefenbachferner (since 2004), Kaunertal (since 2015), and Dachstein (since 2017) do not offer ASTR summer ski anymore. While Pitztal, Rettenbachferner, and Stubai offer (conditions allowing) a small amount of ASTR summer ski due to early ski seasons starts from Mid-September onwards, Kitzsteinhorn offers ASTR summer ski until the end of July but does not reopen until October.

All in all, summer ski operation trends of the Austrian GSAs are rather diverse with relatively high annual fluctuations. In all trend lines, the remarkable drop in the extremely hot summer of 2003 is apparent, which occurred at a time, when summer skiing was still actively pursued by the operators.

**Fig. 2:**
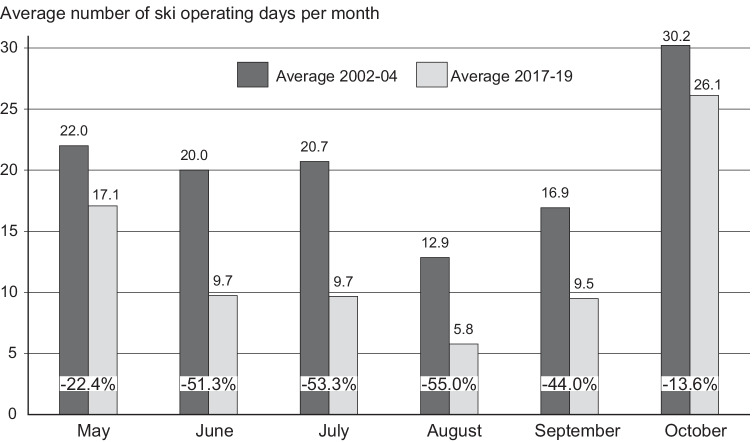
Absolute and relative reduction of monthly ski operating days in Austrian glacier ski areas 2002–2004 compared to 2017–2019 (including Hintertux)

Figure 2, comparing the monthly ski operating day averages of the years 2002–2004 and 2017–2019 (to control for varying weather/snow conditions), shows that the decline of summer ski operation was not evenly spread over the summer: The strongest decline occurred in August (− 55.0%), followed by July (− 53.3%), June (− 51.3%), and September (− 44.0%). The shoulder months May (− 22.4%) and October (− 13.6%) have considerably smaller decreases. Regarding the trend lines of the shares among the potential maximum of operating days per month between 2002 and 2019, a similar picture arises (Supplement 3): October and May have the flattest declining trend lines (− 0.0093 resp. − 0.01), while August (− 0.50), September (− 0.51), and especially June (− 0.68) and July (− 0.80) have the steepest declining trend lines.

### Correlations of summer ski operating days with glaciological and meteorological data

Table 2 shows the correlations of summer ski operating days in Austrian GSAs with (a) meteorological and (b) glaciological data on an aggregated level. Summer mean temperatures at Vernagt meteorological station (R =  − 0.405, *p* < 0.1 to R =  − 0.556, *p* < 0.05) and Hoher Sonnblick observatory (R =  − 0.574, *p* < 0.05 to R =  − 0.744, *p* < 0.001) are negatively correlated with medium to high strength and on varying level of statistical significance with SHY, MET, and ASTR summer ski: the warmer the summers, the less summer ski operation. However, neither sunshine duration in the summer season nor winter snow accumulation or snow depth in summer are significantly correlated with summer ski operation on an aggregated level.

Regarding the glaciological data, the mass balance of Vernagtferner is positively correlated with SHY (R = 0.436, *p* < 0.1), MET (R = 0.379, *p* < 0.2), and ASTR (R = 0.489, *p* < 0.05) operating days at medium strength: This indicates that the less negative the mass balance, the higher the number of summer ski days. However, the mass balance of Stubacher Sonnblickkees and ELA of Vernagtferner are not significantly correlated with the operating days. In contrast, the ELA of Stubacher Sonnblickkees and the AAR of Vernagtferner are significantly related to the number of operating days with the expected signs (ELA: negatively; AAR: positively) and medium strength.

Testing these correlations at the GSA level reveals that the aggregated patterns do not hold true for all destinations (Supplement 4a–c). At best, less than half of the 39 meteorological and glaciological variables are significant (43.6% (17) to 48.7% (19) for Dachstein, Stubai and Rettenbachferner). The respective numbers for Kitzsteinhorn, Pitztal, Kaunertal, and Mölltal are 13 (33.3%), six (15.4%), five (12.8%), and two (5.1%). At Tiefenbachferner, no variable is significant. Interestingly, ASTR summer ski has the highest share of significant correlations (29.8%), especially compared to MET (19.2%). These results indicate that meteorological and glaciological variables are most likely not able to fully explain the trends in the development of summer ski operation in Austria and that their importance varies with seasonality.

### Regression models of summer operating days

#### Yearly/seasonal perspective

First, panel regression models (Tables 3) were applied to test if there is a temporal trend with autoregression eliminated by the Praise-Winsten-procedure. All models have significantly negative time trends.

Second, we added temperature variables to the yearly trend variable: in all models, temperature has a negative influence on the number of summer ski operating days. The additional explanatory power of temperature is relatively limited: it increases by 6.9% (MET), 11.4% (SHY), and 14.1% (ASTR) compared to the time trend only models. Note the higher additional explanatory power of temperature for ASTR vs. MET models.

Third, glacier variables are combined with the time trend. They have a significant influence (signs as expected, see Table 1) on the number of summer ski operating days in all models in which they are included. The additional explanatory power of the glacier variables is higher than those of the temperature variables: it increases by 10.1% (MET), 13.6% (SHY), and 21.7% (ASTR) compared to the time trend only models. Again, the additional explanatory power of glacier variables is much higher for ASTR than MET models.

Next, we looked for models including significant time, temperature and glaciological variables which is only the case for SHY, explaining 34.5% of the variance of summer ski operating days with time having the strongest impact, followed by ELA Vernagtferner and temperature SHY (Table 3, model 4). For MET and ASTR summer ski seasons, temperature is not significant when coupled with glacier variables (models 4 and 8), probably due to multicollinearities between meteorological and glaciological variables, and the explanatory power is slightly lower compared to Model 3 which contains only time trend and glacier variables.

Finally, in the models 5 to 8, we added dummy variables for the GSAs as fixed effect (reference category Mölltal). If added to the time trend, the GSA dummies improve the explanatory power by 30.0% (SHY), 31.8% (MET), and 25.0% (ASTR) (model 5). Including temperature (model 6), glacier variables (model 7), or both (model 8) does improve the models’ explanatory power only slightly: + 3.2% (SHY, model 6), + 0.9% (MET, model 6), and + 2.9% (ASTR, model 7). The highest share of the variance of the summer ski operating days is explained by SHY model 6 (R^2^ adjusted 0.527), MET model 6 (R^2^ adjusted 0.499), and ASTR model 7 (R^2^ adjusted 0.412).

In addition to the panel regression models, we also run individual time series regression models (Table 4) to test if there is a temporal trend for the separate GSA. All GSAs but Mölltal have significantly negative time trends for SHY. For MET and ASTR, Mölltal is significantly positive while Pitztal and Rettenbachferner are not significant and all other remain significantly negative. However, the explanatory power of the time trend varies considerably among the GSAs (between 61.8% Stubai and 8.0% Rettenbachferner).

Adding temperature variables to the yearly trend variable reveals that in 13 out of 24 cases (54.2%) temperature has a significantly negative influence on the number of summer ski operating days. The additional explanatory power of temperature compared to the time trend only models varies a lot between the individual GSAs (average between SHY, MET, and ASTR − 0.9%, Kaunertal, vs. 26.4%, Rettenbachferner; overall average 9.2%).

Next, glacier variables are combined with the time trend. Similar to temperature, in 13 out of 24 cases (54.2%), glacier variables have a significant influence (signs as expected) on the number of summer ski operating days. The additional explanatory power of the glacier variables varies again between the individual GSAs (average between SHY, MET, and ASTR − 2.6%, Kaunertal, vs. 27.2%, Rettenbachferner; overall average 9.7%). Nevertheless, both temperature (mean R^2^ adjusted over 24 models, 0.401; 37.5% of models R adj. > 0.5) and glaciological variables (mean R^2^ adjusted, 0.407; 41.7%) improve the model fit compared to the time trend only models (mean R^2^ adjusted, 0.310; 25.0%).

Finally, we looked for models including significant time, temperature, and glaciological variables. For SHY, we found none. For MET (Table 4), only one model for Kitzsteinhorn significantly combines all three variables at the 10%-level and better, explaining 61.3% of the variance (+ 7.5% compared to the two-variable models). In addition to a negative temperature SHY trend, the mass balance of nearby Stubacher Sonnblickkees positively relates to more summer ski MET days.

Regarding ASTR (Table 4), in three models for Kitzsteinhorn, Tiefenbachferner, and Mölltal, all independent variables significantly influence the operating days. While the Kitzsteinhorn model again improves the two-variable models by 7.8%, its signs are consistent with the MET three-variable model. In contrast, the Mölltal model (explaining 36.5% of the variance) has a positive time sign and a negative AAR of Vernagtferner, indicating that the later in the timeline and the smaller the share of the accumulation area, the higher the number of summer ski ASTR days there - this fits to the descriptive observation but contradicts all considerations about climate change impacts on glacier skiing. Likewise, it is surprising to have the model with the best fit (R^2^ adj. 0.756) for Tiefenbachferner with almost no significant variables in all other models: time trend is negative, AAR Vernagt positive and temperature is also positive, i.e., the warmer the summer, the more summer ski ASTR was possible there - which is highly counterintuitive.

#### Monthly perspective

To provide a more detailed view on the seasonal variation inherent to summer ski operations, we correlated monthly mean Hoher Sonnblick temperatures and snow values with monthly summer ski operating numbers.

**Fig. 3:**
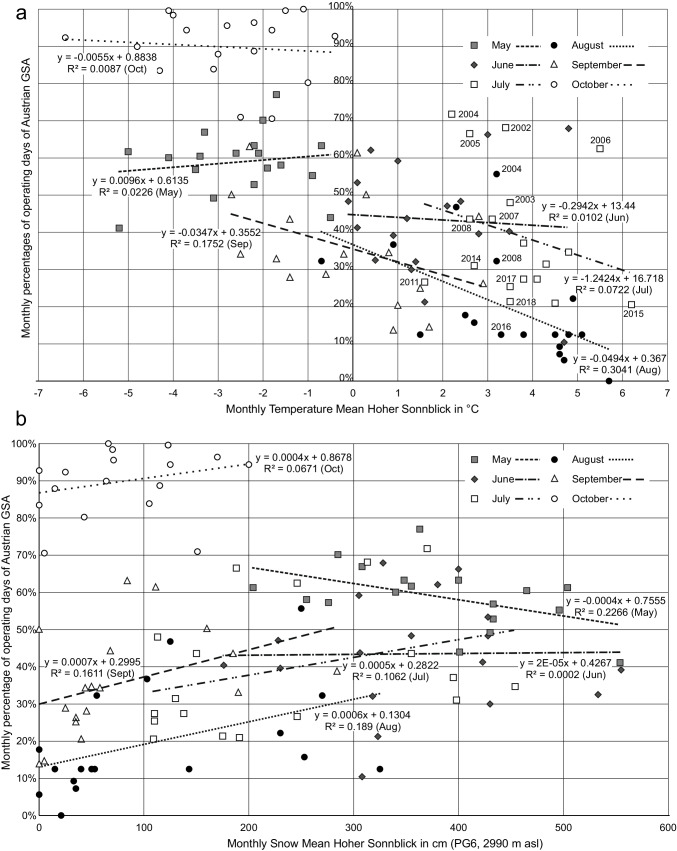
Monthly comparisons between summer ski operating day means of Austrian GSAs and **a** monthly temperature means for Hoher Sonnblick 2002–2019 including some years for July and August values; **b** monthly snow means for Hoher Sonnblick 2002–2019 from PG6 (2990 m asl)

Monthly temperatures and monthly summer ski operating days correlate negatively and highly significantly at a medium to strong level (R =  − 0.673, *p* < 0.001). This is not surprising given the much higher operating day numbers in the cooler months May and October compared with high summer. The correlation just reflects this seasonality but does not indicate anything about effects of global warming. Differentiating this analysis for each month of the summer half-year provides a quite different picture (Fig. 3a): Only August and September show significant negative relations between temperature and operating days. In July, the variance of the mean number of operating days is the highest. For May, June, July, and October, there is no negative correlation between temperature and the number of operating days visible. It is obvious from Fig. 3a that much lower temperatures in May do not induce more operating days than in July in earlier years. It is especially evident for June, July, August, and September that with identical or even lower mean temperatures the share of operating days is considerably lower in later years. This leads to the high variance and not significant regression models. For instance, in 2002, 2003, 2017, and 2018, July had an average temperature at Hoher Sonnblick of 3.4–3.5 °C. While this corresponded to 20.7 operating days (on average) in 2002 and 14.9 in 2003, this decreased to 7.9 (2017) and 6.6 (2018), although the mean temperature was identical.

Regarding the monthly snow depth on Hoher Sonnblick, there is no significant correlation with the monthly mean for the summit observatory (3106 m asl), and the snow measure stations PG6 and PF4. PG3 (2850 m asl, R = 0.181, *p* < 0.1), PG1 (2480 m asl, R = 0.264, *p* < 0.01) and P2 (2710 m asl, R = 0.246, *p* < 0.05) show rather weak positive correlations. Again, this impression changes when analyzing each month separately (Fig. 3b): Although the months July to October show positive relations between snow depth and share of summer ski operating days, the variance remains very high and the explanatory power of linear regression models is very weak to at best rather weak. June shows no trend at all, while May even has a negative relation with the highest R^2^ adjusted value.

Table 5 presents the results of panel regression models for the monthly perspective again using a GSA fixed effect to control for destination variability. The time trend (year) is always statistically significant and negative. The time trend alone has a very weak explanatory power (2.0% of the overall variance, model 1). The monthly mean temperature is statistically significant and negatively related to the share of operating days, except for the models 9 and 10 where its influence is overshadowed by the monthly dummies. Temperature improves the time-only model by 13.7% of explained variance.

Monthly snow depth is statistically significant in all models with a positive sign except for the models 9 and 10 (see above), but with lower explanatory power compared to temperature (improvement of time only-model 1 by only 1.2%). Model 4 includes significant time trend, temperature, and snow variables. However, its overall explanatory power is rather weak (at R^2^ adj. 0.179). In contrast to the yearly models (Table 3), the GSA dummy variables do not explain a relevant share of the operating day variance (10.4%, model 5). Only if they are combined with temperature and snow (models 6, 7), their explanatory power rises (R^2^ adj. 0.266). The explanatory power of the models improves considerably by including the seasonal variation in form of monthly dummies (models 8 to 11) and reach R^2^ adj. 0.390 for the monthly dummies alone (model 8) and culminates at R^2^ adj. 0.440 including the yearly trend, the GSA, and the monthly dummies (model 11). The seasonal variation explains a much higher share of the variance of summer ski operating days compared to time trend, temperature, snow, and GSA dummies. The signs of the monthly dummies are as expected from the operating day trends (Fig. 2): In relation to the reference category “May,” all other summer half-year months have significant negative signs with October significantly deviating to the positive side.

To sum up, there are clear negative time trends for Austrian GSAs. The variation in the summer ski operating days can be partly explained by meteorological and glaciological variables. However, especially the destination level (GSA dummies) and the monthly perspective reveal that a monocausal climate dependence of summer ski operating days is unlikely and, so far, only realistic for high and late summer, while in spring and early summer other factors are more decisive for the reduction of operating days.

### Other factors influencing summer ski operating days

The operators’ agency is a crucial factor. Their strategies and, even more, the selected measures to implement these strategies vary considerably and are summarized in Table 6. A few points must be highlighted.

**Table 6:** Operators’ strategies/decisions regarding summer skiing in Austrian GSAs

Overall direction regarding summer ski	Destination/GSA	Strategy/decisions
a) Continuing of year-around skiing	Hintertux	• Keep all-year round skiing as unique selling proposition in the Eastern Alps (only one other year-round GSA in the Alps, Zermatt) • Considerable adaptation efforts (snowmaking, snowfarming, depots) • Ski operation even under marginal conditions
	Mölltal	• Huge adaptation efforts to sustain main glacier slope: extension of snowmaking system and construction of water reservoir in 2005 • Since 2006 continuous ski operation in eight out of 14 summer seasons • Only GSA with increasing number of ski operating days in the SHY • Attractive location for the south-eastern European market
b) Continuous decline of summer ski operation (focus on May, October and MET summer ski)	Stubai	• Skiing in high summer stopped from 2004 onwards • MET and ASTR summer ski drastically reduced • Less intensive snowmaking on the glacier and fewer depots compared to other GSAs, but extensive glacier covering since early 2000s • Strengthening of non-ski offer in high summer
	Kaunertal	• All-year round skiing stopped in 2001 • ASTR summer ski stopped from 2006 onwards • MET summer ski in continuous decline • Recent trend to large snow depots to open also ski slopes not located on the glacier very early in autumn
	Kitzstein horn	• All-year round skiing stopped after 2006 • Summer ski operation until end of July; ski operation stopped in August and September • Snowmaking along the main glacier slope still not realized • Important role of non-skiing visitors in SHY, especially from Arabian gulf states
c) Complete ending of MET summer ski and ski operation in May (focus on early start in late-summer, autumn)	Rettenbach ferner	• MET summer ski and ski operation in high summer stopped in 2006 • One/two weeks ASTR summer ski due to early season start in September (depending on snow conditions) • Yearly opening of FIS Alpine Ski World Cup end of October as an important event • Ski athletes exclusively use upper part of glacier to exercise even in high summer
	Tiefenbach ferner	• MET and ASTR summer ski stopped from 2003 onwards • SHY ski operation reduced to October and first week of May • Ski athletes exclusively use upper part of glacier to exercise even in high summer
	Pitztal	• Summer ski in high summer already stopped in mid-1990s • Since 2006 no MET summer ski anymore (exception 2021: one week) • Early season start in mid-September leads to usually one week of ASTR summer ski • All-weather-snowmaker and extensive depots allow early opening • Mid-station of highest gondola not renewed in 2012
Shift from b) to c) in 2017	Dachstein	• MET and ASTR summer ski stopped from 2017 onwards (sporadically SHY operation since then) • Continued nordic ski offer until July (unique selling proposition) and general focus on excursionists in summer • Access ropeway often operates at full capacity in the summer season, even without skiers

Source: own compilation

Climate and (directly related) glaciological changes in the GSAs are not always very prominently named by the operators as factors in their decision to reduce summer ski operations. Some of them argue that summer ski operations are primarily demand driven. Only when asked did they admit that in most summers it would no longer be possible to offer summer skiing on their glaciers and that snow conditions had been better in the past. One operator formulated: “We could not satisfy any demand in August, but there was also none”. Thus, while neither climate change nor glacier shrinkage are denied by the operators, some rather argue with commercial considerations leading to the reduction of summer skiing instead of directly blaming climate change influences. Other operators more openly address climate change without being asked for it and name glacier shrinkage as a main challenge (to which they are up to adapt) and one of the reasons for the downturn of summer ski operations (next to changing leisure preferences) is the considerable reduction of skiable terrain in the high summer, i.e., a too small offer. At the Kitzsteinhorn, for instance, the operators reported that summer ski discontinuations until 2011 were always due to lack of snow (for the first time in the 1980s, more often since the 1990s, see Hutter 2005) because the strategy had always been year-around skiing. From 2011 onwards, they deliberately stopped summer skiing in August and September due to a shift of strategy with a focus on excursionists and sightseers in the summer season.

Technical adaptation measures to reduce snow and ice ablation like covering ski slopes and foundations of lift pylons with geotextiles (Fischer et al. 2011, 2016) as well as depot snowmaking to compensate for higher melt rates (Mayer et al. 2007) are widely used. On the one hand, these measures seem to be effective in slowing down the ice melt and preserving snow over the summer which does not need to be artificially produced then (Fischer et al. 2011, 2016) - the best example being the Mölltal GSA which was considered unsuitable for glacier skiing due to its southern exposure and fast shrinking glacier already in the 1980s (Gräbner 1993). On the other hand, some adaptation measures, in particular the covering of the slopes with geotextiles, inhibit the use of the very same slopes for skiing. Given the low demand in late spring and early summer (Kureha 1995; Mayer et al. 2018), it obviously makes more sense for some operators to cover the glaciers and to build snow depots to slow down ice shrinkage and to allow for an early opening in late summer/early autumn when demand by training groups is guaranteed. Consequently, some operators focus on maximizing the overall season length (with the continuation of summer skiing as positive externality of intensive adaptation efforts and strategic positioning), while others concentrate on securing an early start to the winter season in autumn. At Pitztal GSA, the operators even installed the first all-weather snowmaker in 2009 which can produce technical snow above freezing (Mayer et al. 2007; Nöbauer 2018). Also related to technical measures is the temporary closing of summer ski operations due to constructions, mostly of new lifts/ropeways but also of snowmaking installations, which contributes to the high annual fluctuations of operating days (for instance in 2003 for Rettenbachferner,^8^ 2005 for Mölltal and 2015 for Kitzsteinhorn).

The operators are flexible in defining operating times. Summer seasons with better snow conditions are often linked to a higher summer ski supply (as was the case in the relatively cooler summers of 2004 and 2013 - see Fig. 1) and, subsequently, a higher summer ski demand (see Diolaiuti et al. 2006 for an example from Italy). Similarly, some GSAs decided to extend the skiing season 2020/21 into June in the aftermath of the mostly missed winter season due to Covid-19 (Mayer et al. 2021): Pitztal GSA, for instance, offered MET summer ski in 2021 for the first time since 2006.

In some GSAs, glacier ski lifts were replaced by longer, faster and more comfortable gondolas (Mayer 2019). The new transport infrastructure, together with additional attractions at the peak stations (e.g., National park gallery at Kitzsteinhorn), primarily cater to non-skiing visitors. Summer skiing, though, is hampered or becomes even impossible because the bottom station of the gondolas cannot be reached on ski any more due to glacier retreat and/or lack of snow. It can be assumed that the growing number of non-skiers also offers a more promising perspective than the declining number of summer skiers (i.e., excursionists pay similar ticket prices but cause much less costs). Thus, according to some operators, the decision to reduce summer skiing and to focus on non-skiing visitors seems to be perfectly reasonable from a financial point of view.

Furthermore, the end of year-around skiing provided operators, staff and the destinations as a whole with a much desired seasonal break, necessary to recover from strenuous and extended “winter” seasons. The social toll of these - from the tourism actors’ subjective perspective - seemingly never-ending seasons on the destinations and their managing and operating staff in GSAs, accommodation, and gastronomy has been reported already by Hupke (1990).

Finally, there is a marketing perspective. Year-round skiing was often used to highlight the area’s winter season snow reliability. Some GSAs offered “alibi summer skiing” (i.e., one ski slope serviced by a single ski lift, even under marginal conditions) to keep this marketing claim alive. However, this marketing argument continually lost importance with the diffusion of technical snowmaking (Mayer 2012). Further, we assume that pictures of questionable summer ski offers in today’s social media would likely lead to negative debates and potentially damage the areas’ image - this assumption should be subject of further research. Interestingly, the webcams of most GSAs are either switched off or turned away from the ski slopes in high summer to avoid customers seeing the glaciers in bad conditions (Nadegger 2020).

All in all, the operators’ decision to reduce summer ski operation in most Austrian GSAs can be characterized as a complex nexus of a shifted focus on seasons (surely also due to climate change and glacier shrinkage), negative externalities of adaptation measures, and a demand shortage coupled with rising operating costs and lost marketing appeal.

### Discussion and conclusions

Our results reveal a negative relationship between global warming and summer ski supply in Austria between 2002 and 2019. For instance, the less negative the glacier mass balances, which indicates better skiing conditions, the higher the number of ski operating days in summer. The regression models, though, also indicate that the decline of summer ski supply can only partly be explained by meteorological and glaciological data. There is, for example, no negative correlation between monthly temperatures (except for August and September) and the number of operating days.^9^ Sunshine duration in the summer season, winter snow accumulation, and summer snow depth are also not significantly correlated with summer ski operating days on an aggregated level. Regarding sunshine duration, the meteorological station might to be too far away from most GSAs to offer relevant data. In the case of Kitzsteinhorn, which is relatively close to the Sonnblick meteorological station, there is a negative correlation between sunshine duration and the MET and ASTR summer ski seasons (see Supplements 4b and c). Changes in the mass balance of glaciers are primarily driven by the summer temperatures in the Alps (Vincent et al. 2005); thus, it is not surprising that winter snow accumulation does not affect summer ski supply. With respect to snow depth in summer, the missing relation is less obvious and might be explained by too less site-specific data - snow depths vary a lot (e.g., wind accumulation, exposition) and can be further affected by the operators’ decision to actively manage/farm snow. Thus, similar to the situation in winter where the snow depth is influenced by snowmaking, the snow depth on the summer ski slopes could be detached from the natural snow conditions as well.

The notable difference in the ski offers between the months May and June (sharp decrease of monthly ski operations) and the months September and October (sharp increase of monthly ski operations (Fig. 2) clearly shows that the snow conditions do not solely explain the decline in summer ski operation as the late spring/early summer is usually not hampered by a lack of snow but by a lack of demand - for late summer/early autumn exactly the opposite holds true (Mayer et al. 2018). This mismatch of glacier ski supply and demand was already pointed out by Barnick (1970) and Mayer (2012). The monthly panel regression models (Table 5) show that the monthly dummies explain much higher shares of the overall variance than the temperature and snow variables. As the monthly dummies also include the seasonal variation of the temperature and snow variables, the higher explanatory power could be interpreted as covering the otherwise undetected variability brought in by the operators’ decisions whether or not to open summer skiing in the respective months.

Strictly speaking, there is no linear decline in summer ski operating days as there are still summer seasons with better snow conditions, e.g., the relatively cool summers of 2004 and 2013 vs. the warmer summers of 2003 and 2012.^10^ Better conditions in cooler summers are often linked to a higher summer ski supply (Fig. 1). This was confirmed by Diolaiuti et al. (2006) who reported both supply and demand flexibility, i.e., more summer skiers benefiting from better skiing opportunities in cooler and wetter summers. Another sign of the operators’ flexibility is their summer ski offer for athletes only - in GSAs which have abandoned public MET/ASTR summer skiing.

It is the operators’ agency (materialized in their policies, strategies, investments, etc.) that was/is crucial for the summer ski operation in Austrian GSAs. While the challenges of global warming are similar to all GSAs, the way how the operators deal with them varies considerably. Depending on the operators’ adaptation efforts, it was possible to counteract glacier shrinkage and to partly replace the melted-off parts of former glacier slopes with artificial snow/ice bands - at least in the last 10 years. GSAs such as Hintertux and Mölltal showed that the extensive implementation of technical adaptation measures can lead to continued summer ski operations. Other GSAs focused on the safeguarding of an early start into the new autumn/winter season leading to some days of ASTR summer skiing in September. The adaption measures also guarantee ski operation in October which is considered to be a unique selling proposition for GSAs. Technical adaptation measures, thus, are crucial, not only for the winter but also for the summer ski season (Fischer et al. 2016). Mölltal GSA showcases the paradoxical situation that a GSA originally deemed unsuitable for summer skiing is the only Austrian GSA to extend MET and ASTR summer ski operating days since 2002.

Our research, however, is also subject to limitations: First, due to lacking meteorological and glaciological data for individual GSAs, we had to work with proxies, although local data would surely be more precise. Second, operating days do not state anything about skiable slope surface, number of lifts in operation, quality of skiing, and demand/turnover. Third, delay and long-term effects of global warming are not considered yet. The ongoing glacier melt has long-time repercussions for ski operations and on the operators’ perspectives on climate change and summer skiing. These effects could be compared to a bank account which runs a deficit for years: the substance of the portfolio is decreasing continuously although the running costs can still be paid, but at some point in time the account is empty. In the glacier skiing context, this analogy means that areas get finally ice-free which can lead to considerable difficulties for the ski operation although glacier downwasting began already decades earlier. These cumulative, long-term effects of global warming might lead to the stop of summer ski operation, but they cannot be explained sufficiently by the yearly meteorological and glaciological data as they would require much more complex modeling.

To conclude, between 2002 and 2019, summer ski operating days in Austrian GSAs declined by 48.3% in the SHY, 65.2% in the MET, and 62.3% in the ASTR summer ski seasons, among other driven by increasing temperatures and shrinking glaciers. The bandwidth of these results underlines that summer ski decline figures depend to a considerable degree on the definition used for summer skiing. This also holds true for its future perspectives: ASTR summer skiing including the first 3 weeks of September will become more difficult to maintain without the extensive use of adaptation measures such as snowfarming and all-weather snowmakers. In contrast, MET summer skiing including the first 3 weeks of June will be more easily to maintain as winter snow in high alpine areas should (normally) be abundant enough to allow for skiing in June and, potentially, in the first half of July. Thus, the influences of global warming on summer ski operating days must be differentiated seasonally: the influences are strongest in high and late summer, while in spring and early summer the decline is (currently) mostly influenced by a lack of demand and the operators’ decision to save costs by shutting down the lifts.

The decline of summer ski operation is an indication of progressing climate change. Areas, where people previously used to ski in high summer, are now characterized by unsuitable conditions or completely ice-free. It needs to be emphasized, though, that the decline in summer ski operating days is not one-dimensionally caused by global warming. Even in the presence of strong climatic signals, it is the human agency of the operators that has played a crucial role for the development of the GSAs - at least up to now. The full impact of climate change, however, is difficult to determine: Several of the non-climatic factors influencing the decline of summer ski operating days identified in this study cannot be wholly understood without considering the changing climatic conditions and, thus, could be understood as indirect effects of climate change. It is likely that the attractivity loss, the fall and seasonal shift of demand, the rise of operating costs, and subsequently the reassessment of summer skiing as a commercial activity would have happened without climate change. But it is also likely that these developments (and the operators’ decisions behind these developments) have been affected and reinforced by the changing climate conditions (e.g., worsening snow conditions in summer reduced attractivity and demand). This calls for a systemic research approach bearing in mind that the evolution of GSAs is influenced by a series of interacting climatic and non-climatic factors.

In future research, daily operation, weather, and snow data could be combined with demand data. This would help to further analyze the supply–demand mismatch in glacier skiing. Further, it would be interesting to investigate the offers which have been implemented to complement or replace the summer ski operation in the Austrian GSAs. Mayer et al. (2018) and Carver and Tweed (2021) provided some information about the Kitzsteinhorn and Stubai GSAs but an in-depth analysis is missing. In addition, compared to glacier visitors outside GSAs (Salim et al. 2022), the climate change perception of glacier and summer skiers in the Alps has not been investigated yet. Finally, future contributions should broaden the spatial and temporal scales by researching the development of glacier and summer skiing in other Alpine countries as well as by reconstructing this development much further back into the past. Using both physical (i.e., climate, glacier, snow, e.g., Olefs et al. 2020) and socio-economic scenarios, the future of GSAs could also be modeled.

## Supplementary Information

Below is the link to the electronic supplementary material.

ESM 1 (PDF 819 KB)

## Data Availability

Not applicable**.**

## Footnotes

1 Glacier ski supply is also positively correlated to skier days. Analyses for Kitzsteinhorn GSA (based on Mayer et al. 2018) show a strong positive correlation of R 0.78^***^ between the operating lift capacity and the skier days on a daily basis (November 1, 2005–April 30, 2013, *n* = 2738).

2 Between May 1 and October 31, there are 184 days. Times nine summer ski areas (SSA) this leads to 1656 days of aggregated potential ski operating days in the summer half year. For MET summer ski, this potential maximum is 92 days times nine SSA = 828 days. For ASTR summer ski, a maximum of 93 days times nine SSA sums up to 837 days. The varying length of the summer months (June and September 30 days, the rest 31) was corrected by using percentages.

3 This use of proxy data can be justified by the results of Koch et al. (2020). They modeled the snow situation for Obergurgl ski area which reaches an altitude of 3000 m using Snowgrid-CL. As there are no meteorological stations in these altitudes for validation they compared the snow gauge measurements from Hoher Sonnblick with the modeled snow levels for Obergurgl and could not find relevant deviations (Fig. 29, p. 38).

4 We used the data of different snow measurement stations in different altitudes at the Hoher Sonnblick to test for the influences of altitude and location at/near a glacier. For the following stations gauge measurements are used: PG1: Unterer Goldbergkeesboden 2480 m asl; PG2: Oberer Goldbergkeesboden 2710 m asl; PG3: Goldbergkees Oberer Steilhang 2850 m asl; PG6: Fleißscharte Goldbergkees 2990 m asl; PF4: Pilatusscharte Fleißkees 2880 m asl.; the daily snow values of Hoher Sonnblick 3109 m asl. are derived from laser measurements.

5 The accumulation area ratio (AAR) indicates the ratio of the accumulation area to the total glacier area in percent. As a rule of thumb, the mass balance of a glacier is balanced when the ratio of the size of the accumulation area to that of the ablation area is about 2:1 (AAR = 66%). (Bayerische Akademie der Wissenschaften 2021b).

6 The equilibrium line altitude (ELA) is a hypothetical quantity determined on the basis of the altitude distribution of the specific net balance of a glacier. By this altitude line the glacier is divided into two areas: above are the areas with a net mass gain, below there is a mass loss (Bayerische Akademie der Wissenschaften 2021b).

7 Hintertux is a special case as it is the only Austrian GSA still offering skiing all year around, so operating days there did not change. Therefore, its operating days were neither included into the correlation analyses nor the regression models.

8 To compensate for this closure, the neighboring Tiefenbachferner had the highest number of MET and ASTR summer ski operating days of the whole observation period in this extremely warm summer of 2003 which turned out to be the last MET and ASTR summer ski season there.

9 This is probably due to the fact that in the early 2000s summer skiing was still offered at much higher temperatures (see the four July values with high shares of operating days > 70% or just below in Fig. 3a), which was either obviously possible from the snow/glacier situation (snow sufficient but of, most likely, very slushy quality) or was operationally desired (for marketing reasons, see below) or tolerated (only few skiers and conditions are not well, but we keep it open).

10 Hoher Sonnblick mean temperature April to September: 0.6 °C (2003) vs. − 1.0 °C (2004); SHY operating days 55.6% (2003) vs. 71.0% (2004); 0.5 °C (2012) vs. − 0.1 °C (2013); SHY operating days 44.1% (2012) vs. 52.3% (2013).
