# Supplementary material for: Development of summer skiing days in Austrian glacier ski areas in the first two decades of the twenty-first century
Source: Int J Biometeorol. 2022 Oct 1;68(3):547–64. doi: 10.1007/s00484-022-02371-6 (PMC9525926; doi:10.1007/s00484-022-02371-6)
Supplement: Supplementary file 1 — (PDF 819 KB) [file 484_2022_2371_MOESM1_ESM.pdf]

## Supplementary materials to "Development of summer skiing days in Austrian glacier ski areas in the first two decades of the 21st century"

### Supplement 1. Characteristics of Austrian glacier ski areas (2019), sorted by ski area size measured in lift capacity

| Name | Year opening glacier skiing | Number of lifts in glacier ski area | Number of lifts in rest of ski area | Lift capacity in million VTMH corrected with glacier share of lift length | Altitude max. in m asl | Altitude difference max. to lowest part of glacier in m |
|---|---|---|---|---|---|---|
| Hintertux | 1969 | 11 | 8 | 6.899 | 3250 | 625 |
| Stubai | 1973 | 11 | 10 | 4.527 | 3210 | 440 |
| Pitztal | 1983 | 4 | 7 | 2.403 | 3428 | 533 |
| Kitzsteinhorn | 1966 | 7 | 7 | 2.388 | 2926 | 317 |
| Rettenbachferner | 1975 | 4 | 30 | 2.198 | 3247 | 573 |
| Mölltal | 1987 | 3 | 5 | 1.437 | 3089 | 362 |
| Tiefenbachferner | 1981 | 2 | 30 | 1.343 | 3249 | 329 |
| Kaunertal | 1980 | 5 | 2 | 1.034 | 3107 | 397 |
| Dachstein | 1969 | 3 | 1 | 0.317 | 2694 | 167 |

VTMH: Vertical Transport Meter Per Hour

Source: own compilation based on technical data of the operators/lift-world.info, own measurements in aerial photographs

## Supplement 2a-c: Operating day trends for all Austrian GSA except Hintertux

### 2a: SHY

| | Hinter-tux | Stubai | Kaunertal | Kitzstein-horn | Rettenbach-ferner | Tiefenbach-ferner | Pitztal | Mölltal | Dach-stein | Aggregated | In percent of maximum |
|---|---|---|---|---|---|---|---|---|---|---|---|
| 2002 | 184 | 163 | 133 | 172 | 85 | 145 | 70 | 106 | 159 | 1217 | 73.5% |
| 2003 | 184 | 115 | 129 | 123 | 33 | 99 | 74 | 97 | 67 | 921 | 55.6% |
| 2004 | 184 | 173 | 136 | 184 | 138 | 40 | 78 | 107 | 135 | 1175 | 71.0% |
| 2005 | 184 | 130 | 128 | 184 | 135 | 47 | 70 | 79 | 167 | 1124 | 67.9% |
| 2006 | 184 | 117 | 105 | 184 | 59 | 34 | 83 | 153 | 151 | 1070 | 64.6% |
| 2007 | 184 | 108 | 92 | 162 | 54 | 33 | 67 | 124 | 133 | 957 | 57.8% |
| 2008 | 184 | 118 | 81 | 159 | 47 | 26 | 66 | 133 | 144 | 958 | 57.9% |
| 2009 | 184 | 88 | 62 | 153 | 47 | 20 | 72 | 143 | 88 | 857 | 51.8% |
| 2010 | 184 | 88 | 88 | 140 | 54 | 32 | 63 | 136 | 62 | 847 | 51.1% |
| 2011 | 184 | 98 | 81 | 108 | 42 | 22 | 46 | 139 | 89 | 809 | 48.9% |
| 2012 | 184 | 88 | 61 | 99 | 45 | 25 | 33 | 122 | 73 | 730 | 44.1% |
| 2013 | 184 | 108 | 88 | 116 | 47 | 33 | 60 | 145 | 85 | 866 | 52.3% |
| 2014 | 184 | 114 | 82 | 94 | 58 | 33 | 54 | 146 | 83 | 848 | 51.2% |
| 2015 | 184 | 101 | 68 | 42 | 54 | 33 | 53 | 144 | 70 | 749 | 45.2% |
| 2016 | 184 | 61 | 61 | 116 | 53 | 26 | 54 | 144 | 89 | 788 | 47.6% |
| 2017 | 184 | 96 | 76 | 116 | 60 | 33 | 53 | 123 | 94 | 835 | 50.4% |
| 2018 | 184 | 60 | 67 | 103 | 30 | 8 | 47 | 134 | 6 | 639 | 38.6% |
| 2019 | 184 | 90 | 46 | 76 | 54 | 35 | 46 | 96 | 2 | 629 | 38.0% |
| 2002-2019 in percent | 0.0% | -44.8% | -65.4% | -55.8% | -36.5% | -75.9% | -34.3% | -9.4% | -98.7% | -48.3% | -48.3% |
| 2002-2019 absolute values | 0 | -73 | -87 | -96 | -31 | -110 | -24 | -10 | -157 | -588 | |
| Mean 2000s | 184.0 | 126.5 | 108.3 | 165.1 | 74.8 | 55.5 | 72.5 | 117.8 | 130.5 | 1034.9 | 62.5% |
| Mean 2010s | 184.0 | 90.4 | 71.8 | 101.0 | 49.7 | 28.0 | 50.9 | 132.9 | 65.3 | 774.0 | 46.7% |
| Percentual Change means 2000s-2010s | 0.0% | -28.5% | -33.7% | -38.8% | -33.5% | -49.5% | -29.8% | 12.9% | -50.0% | -25.2% | -25.2% |

**SHY:** As for 2019, three groups of GSA except for Hintertux can be identified: While Dachstein almost eliminated SHY operation, four GSA (Rettenbach- and Tiefenbachferner, Kaunertal and Pitztal) offer between 35 and 54 days (i.e. between one and nearly two months), while three GSA (Kitzsteinhorn, Stubai and Mölltal) operated on 76 to 96 days (two and a half to more than three months). The strongest absolute and relative declines occurred at Dachstein (-157 days, -98.7%), Tiefenbachferner (-110 days, -75.9%), Kaunertal (-87 days, -65.4%), Kitzsteinhorn (-96 days, -55.8%), Stubai (-73 days, -44.8%), while Rettenbachferner (-31 days, -36.5%) and Pitztal (-24 days, -34.3%) show less intense decline – due to the lower starting level in 2002. However, taking 2004 with the highest number of operating days as a starting point for Rettenbachferner, the decline also amounts to -60.9%.

## Supplement 2b: MET

| | Hinter-tux | Stubai | Kaunertal | Kitzstein-horn | Rettenbach-ferner | Tiefenbach-ferner | Pitztal | Mölltal | Dach-stein | Aggregated | In percent of maximum |
|---|---|---|---|---|---|---|---|---|---|---|---|
| 2002 | 92 | 87 | 54 | 80 | 12 | 80 | 0 | 45 | 86 | 536 | 64,7% |
| 2003 | 92 | 42 | 56 | 48 | 0 | 45 | 1 | 44 | 46 | 374 | 45,2% |
| 2004 | 92 | 92 | 53 | 92 | 68 | 0 | 0 | 66 | 74 | 537 | 64,9% |
| 2005 | 92 | 61 | 54 | 92 | 72 | 0 | 0 | 33 | 90 | 494 | 59,7% |
| 2006 | 92 | 54 | 51 | 92 | 25 | 0 | 5 | 78 | 79 | 476 | 57,5% |
| 2007 | 92 | 30 | 21 | 77 | 0 | 0 | 0 | 61 | 76 | 357 | 43,1% |
| 2008 | 92 | 45 | 15 | 92 | 0 | 0 | 0 | 73 | 79 | 396 | 47,8% |
| 2009 | 92 | 14 | 14 | 79 | 0 | 0 | 0 | 73 | 66 | 338 | 40,8% |
| 2010 | 92 | 12 | 13 | 69 | 0 | 0 | 0 | 66 | 43 | 295 | 35,6% |
| 2011 | 92 | 26 | 12 | 55 | 0 | 0 | 0 | 70 | 39 | 294 | 35,5% |
| 2012 | 92 | 17 | 10 | 52 | 0 | 0 | 0 | 62 | 45 | 278 | 33,6% |
| 2013 | 92 | 35 | 16 | 51 | 0 | 0 | 0 | 72 | 51 | 317 | 38,3% |
| 2014 | 92 | 34 | 9 | 34 | 0 | 0 | 0 | 74 | 51 | 294 | 35,5% |
| 2015 | 92 | 33 | 7 | 0 | 0 | 0 | 0 | 73 | 46 | 251 | 30,3% |
| 2016 | 92 | 0 | 0 | 54 | 0 | 0 | 0 | 75 | 42 | 263 | 31,8% |
| 2017 | 92 | 18 | 5 | 53 | 0 | 0 | 0 | 61 | 37 | 266 | 32,1% |
| 2018 | 92 | 3 | 3 | 52 | 0 | 0 | 0 | 77 | 0 | 227 | 27,4% |
| 2019 | 92 | 10 | 0 | 25 | 0 | 0 | 0 | 60 | 0 | 187 | 22,6% |
| 2002-2019 in percent | 0.0% | -88.5% | -100.0% | -68.8% | -100.0% | -100.0% | - | 33.3% | -100.0% | -65.1% | -65.1% |
| 2002-2019 absolute values | 0 | -77 | -54 | -55 | -12 | -80 | 0 | 15 | -86 | -349 | |
| Mean 2000s | 92.0 | 53.1 | 37.7 | 81.5 | 23.6 | 6.4 | 0.9 | 59.1 | 74.5 | 438.5 | 53.0% |
| Mean 2010s | 92 | 18.8 | 7.5 | 44.5 | 0 | 0 | 0 | 69 | 35.4 | 267.2 | 32.3% |
| Percentual Change means 2000s-2010s | 0.0% | -64.6% | -80.1% | -45.4% | -100.0% | -100.0% | -100.0% | 16.7% | -52.5% | -39.1% | -39.1% |

**MET summer ski:** While Hintertux remains constant and Mölltal increases its operating days (+15 days, +33.3%), Pitztal offered no MET summer ski throughout (with two exceptions), Tiefenbach- and Rettenbachferner stopped MET summer ski from 2003 and 2006 onwards respectively, Kaunertal reached zero MET summer ski days in 2016 and 2019 after a long agony since 2007 with less than three weeks of MET summer ski operation. Kitzsteinhorn in contrast remained on a very high level until 2008 (92 days, 100% operation in four years since 2002) and started a relatively linear decline then, leading to 25 days in 2019 (-68.8%). Dachstein similarly left the high level of MET summer ski operation after 2008 and stopped it completely after 2017. Finally, Stubai also started with nearly full MET summer ski operation, followed by a sharp decline until 2010 (only 12 days left), but also showed a brief resurgence (between 2013 and 2015 up to 35 days) and then further decline (10 days in 2019, -88.5% compared to 2002).

## Supplement 2c: ASTR

| | Hinter-tux | Stubai | Kaunertal | Kitzstein-horn | Rettenbach-ferner | Tiefenbach-ferner | Pitztal | Mölltal | Dach-stein | Aggregated | In percent of maximum |
|---|---|---|---|---|---|---|---|---|---|---|---|
| 2002 | 93 | 72 | 42 | 85 | 33 | 60 | 8 | 32 | 89 | 514 | 61,4% |
| 2003 | 93 | 24 | 38 | 32 | 0 | 34 | 2 | 32 | 32 | 287 | 34,3% |
| 2004 | 93 | 84 | 45 | 93 | 88 | 0 | 7 | 55 | 93 | 558 | 66,7% |
| 2005 | 93 | 41 | 37 | 93 | 79 | 0 | 7 | 27 | 89 | 466 | 55,7% |
| 2006 | 93 | 34 | 31 | 93 | 25 | 0 | 7 | 93 | 79 | 455 | 54,4% |
| 2007 | 93 | 28 | 1 | 71 | 8 | 0 | 7 | 64 | 67 | 339 | 40,5% |
| 2008 | 93 | 27 | 0 | 72 | 3 | 0 | 8 | 81 | 62 | 346 | 41,3% |
| 2009 | 93 | 3 | 0 | 62 | 3 | 0 | 8 | 93 | 46 | 308 | 36,8% |
| 2010 | 93 | 5 | 4 | 49 | 12 | 0 | 7 | 87 | 34 | 291 | 34,8% |
| 2011 | 93 | 7 | 0 | 35 | 0 | 0 | 0 | 91 | 19 | 245 | 29,3% |
| 2012 | 93 | 0 | 0 | 32 | 0 | 0 | 0 | 69 | 25 | 219 | 26,2% |
| 2013 | 93 | 17 | 1 | 31 | 2 | 0 | 8 | 93 | 31 | 276 | 33,0% |
| 2014 | 93 | 23 | 2 | 25 | 14 | 0 | 2 | 93 | 31 | 283 | 33,8% |
| 2015 | 93 | 13 | 0 | 0 | 10 | 0 | 3 | 93 | 26 | 238 | 28,4% |
| 2016 | 93 | 0 | 0 | 34 | 5 | 0 | 6 | 93 | 23 | 254 | 30,3% |
| 2017 | 93 | 6 | 0 | 33 | 13 | 0 | 6 | 65 | 19 | 235 | 28,1% |
| 2018 | 93 | 0 | 0 | 32 | 0 | 0 | 7 | 89 | 0 | 221 | 26,4% |
| 2019 | 93 | 9 | 0 | 23 | 9 | 0 | 1 | 59 | 0 | 194 | 23,2% |
| 2002-2019 in percent | 0.0% | -87.5% | -100.0% | -72.9% | -72.7% | -100.0% | -87.5% | 84.4% | -100.0% | -62.3% | -62.3% |
| 2002-2019 absolute values | 0 | -63 | -42 | -62 | -24 | -60 | -7 | 27 | -89 | -320 | |
| Mean 2000s | 93.0 | 39.1 | 21.7 | 75.1 | 29.4 | 4.9 | 6.6 | 59.6 | 69.6 | 394.1 | 47.1% |
| Mean 2010s | 93.0 | 8.0 | 0.7 | 29.4 | 6.5 | 0.0 | 4.0 | 83.2 | 20.8 | 245.6 | 29.3% |
| Percentual Change means 2000s-2010s | 0.0% | -79.6% | -96.8% | -60.9% | -77.9% | -100.0% | -39.1% | 39.5% | -70.1% | -37.7% | -37.7% |

**ASTR summer ski**: The special case of Mölltal is even more obvious with an increase of 84.4% between 2002 and 2019 (from 32 to 59 days); also the average of the 2010s is 39.5% higher than the one of the 2000s. Tiefenbachferner (since 2004), Kaunertal (since 2015) and Dachstein (since 2017) do not offer ASTR summer ski anymore. Pitztal usually starts the ski season in Mid-September, so they offer between 6.6 days (average 2000s) and 4.0 days (average 2010s, -39.1%) of ASTR summer ski in September. Rettenbachferner adopted this operation policy from 2007 onwards (6.1 ASTR summer ski days average 2007-19 in September), in contrast to 2004 and 2005 when nearly the complete ASTR summer ski season was covered (88 resp. 79 days). Stubai almost offered a complete ASTR summer ski season for the last time in 2004 (84 days), followed by a sharp decline until 2009 (three days). Since then, the ASTR summer ski operation varies and amounts in the 2010s to eight days on average (-87.5% between 2002 and 2019). Kitzsteinhorn offered a complete ASTR summer ski season for the last time in 2006 and shows a linear decline until 2011 (from 93 to 35 days); since then, the level of ASTR summer ski operation is rather stable (23 in 2019, -72.9% since 2002) and the third longest time span after Hintertux and Mölltal. Its trend is rather similar to the Dachstein (complete ASTR summer ski season in 2004, sharp decline until 2011, stable level until the stop after 2017).

**Supplement 3: Development of summer ski operating days on a monthly level 2002-2019 (with Hintertux)**

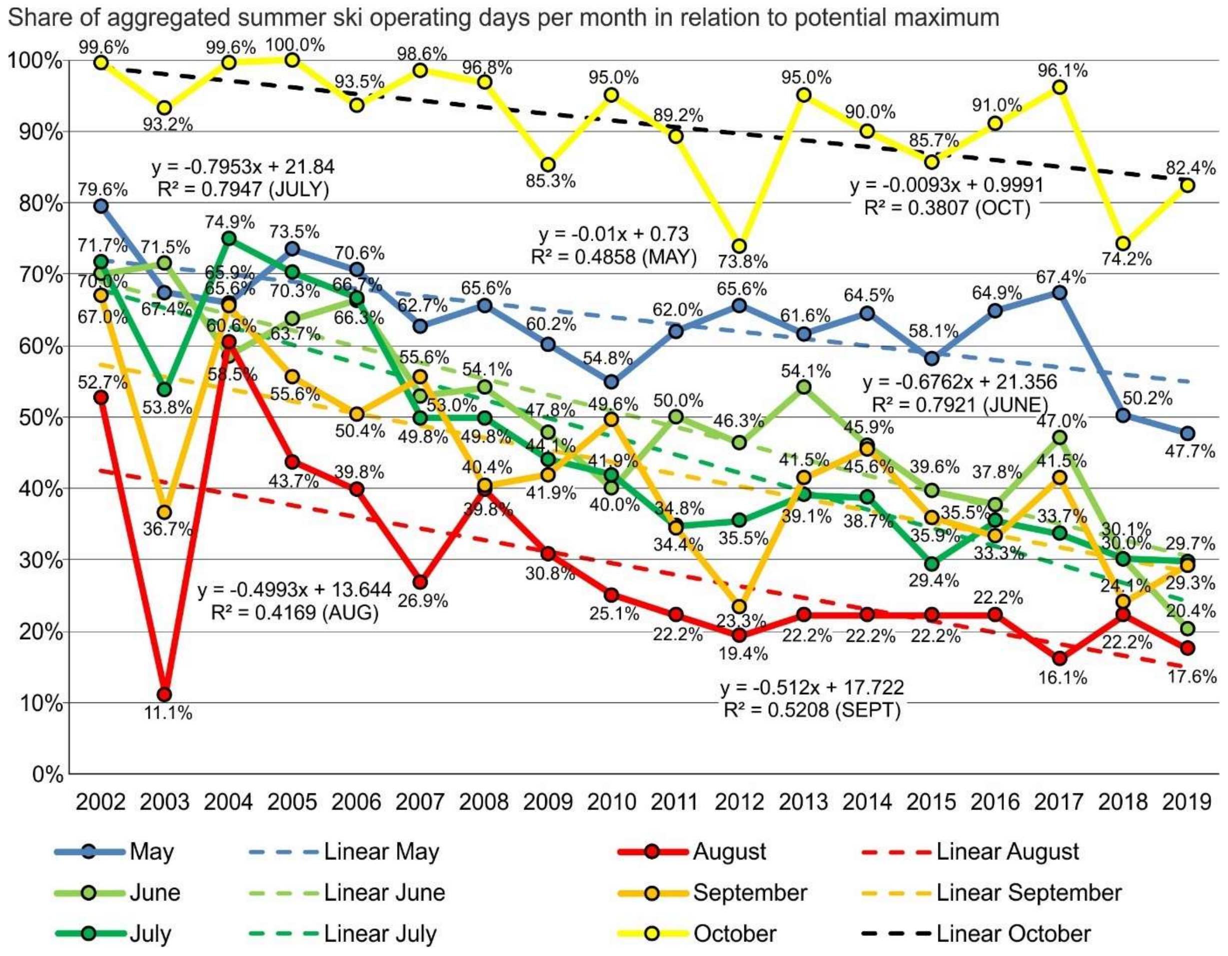

**Supplement 4a. Correlations of summer ski operating days with meteorological and glaciological data: GSA level, Summer half-year**

| | Stubai | Kaunertal | Kitzstein-horn | Retten-bachferner | Tiefen-bachferner | Pitztal | Mölltal | Dach-stein |
|---|---|---|---|---|---|---|---|---|
| Vernagtferner Temp. Summer (Jun-Sept) | **-0.494*** | **-0.402#** | **-0.414#** | **-0.491*** | -0.271 | -0.359 | 0.043 | **-0.527*** |
| Hoher Sonnblick Temp. Summer (Jun-Aug) | -0.383 | **-0.402#** | **-0.643**** | **-0.528*** | 0.154 | **-0.430#** | -0.188 | **-0.636**** |
| Hoher Sonnblick Temp. April-Sept | **-0.723**** | **-0.578*** | **-0.561*** | **-0.681**** | -0.239 | **-0.528*** | 0.116 | **-0.721**** |
| Hoher Sonnblick Sunshine duration Summer (Jun-Aug) | -0.050 | -0.045 | -0.356 | -0.224 | 0.204 | 0.073 | -0.099 | -0.333 |
| Vernagtferner mean snow depth (May 1) converted to water equivalent mm | 0.034 | -0.183 | -0.306 | -0.218 | 0.150 | -0.127 | 0.027 | -0.350 |
| Hoher Sonnblick mean snow depth (May 1) | -0.031 | -0.197 | -0.190 | -0.204 | 0.071 | 0.207 | 0.240 | -0.139 |
| Hoher Sonnblick mean snow depth SHY | 0.009 | -0.122 | -0.132 | 0.073 | -0.211 | 0.185 | **0.404#** | -0.078 |
| Vernagtferner mass balance | **0.436#** | 0.189 | 0.341 | **0.534*** | 0.048 | 0.161 | 0.095 | **0.437#** |
| Stubacher Sonnblick-kees mass balance | 0.188 | -0.077 | 0.287 | 0.335 | -0.205 | 0.109 | 0.139 | 0.219 |
| Vernagtferner ELA | -0.281 | -0.047 | -0.112 | **-0.401#** | 0.050 | 0.077 | -0.284 | -0.355 |
| Stubacher Sonnblick-kees ELA | **-0.428#** | -0.241 | **-0.430#** | **-0.527*** | 0.038 | -0.378 | -0.123 | -0.383 |
| Vernagtferner AAR | **0.568*** | 0.377 | 0.271 | **0.553*** | 0.334 | 0.185 | -0.088 | **0.431#** |
| Stubacher Sonnblick-kees AAR | **0.435#** | 0.252 | 0.306 | **0.472*** | 0.036 | 0.335 | 0.042 | 0.261 |
| Nr of sig. variables (max 13) | 6 | 3 | 4 | 8 | 0 | 2 | 1 | 5 |

**Supplement 4b: Correlations of summer ski operating days with glaciological and meteorological data: GSA level, summer ski MET**

| | Stubai | Kaunertal | Kitzstein-horn | Rettenbach-ferner | Tiefenbach-ferner | Pitztal | Mölltal | Dach-stein |
|---|---|---|---|---|---|---|---|---|
| Vernagtferner Temp. Summer (Jun-Sept) | -0.398 | -0.195 | -0.332 | -0.245 | -0.143 | 0.248 | 0.183 | **-0.530*** |
| Hoher Sonnblick Temp. Summer (Jun-Aug) | **-0.430#** | -0.339 | **-0.645**** | **-0.566*** | 0.219 | -0.227 | -0.121 | **-0.604**** |
| Hoher Sonnblick Temp. April-Sept | **-0.682**** | **-0.468#** | **-0.484*** | **-0.555*** | -0.061 | -0.113 | 0.187 | **-0.707**** |
| Hoher Sonnblick Sunshine duration Summer (Jun-Aug) | -0.093 | -0.019 | **-0.441*** | -0.246 | 0.159 | -0.068 | -0.094 | -0.245 |
| Vernagtferner mean snow depth (May 1) converted to water equivalent mm | 0.041 | -0.083 | -0.294 | -0.200 | 0.257 | -0.057 | 0.195 | -0.282 |
| Hoher Sonnblick mean snow depth (May 1) | -0.040 | -0.184 | -0.209 | -0.285 | 0.100 | 0.007 | 0.320 | -0.058 |
| Hoher Sonnblick mean snow depth MET summer | 0.022 | -0.080 | -0.182 | 0.019 | -0.187 | -0.039 | 0.344 | 0.008 |
| Vernagtferner mass balance | **0.403#** | 0.095 | 0.315 | 0.366 | -0.033 | -0.124 | 0.068 | **0.425#** |
| Stubacher Sonnblick-kees mass balance | 0.181 | -0.084 | 0.354 | 0.267 | -0.228 | -0.066 | 0.267 | 0.219 |
| Vernagtferner ELA | -0.288 | 0.086 | -0.109 | -0.226 | 0.089 | 0.142 | -0.214 | -0.292 |
| Stubacher Sonnblick-kees ELA | **-0.417#** | -0.216 | **-0.404#** | **-0.466#** | 0.106 | -0.008 | -0.135 | **-0.443#** |
| Vernagtferner AAR | **0.541*** | 0.284 | 0.181 | 0.387 | 0.236 | -0.061 | -0.172 | **0.419#** |
| Stubacher Sonnblick-kees AAR | 0.397 | 0.216 | 0.250 | **0.407#** | -0.053 | -0.026 | 0.071 | 0.337 |
| Nr of sig. variables (max 13) | 5 | 1 | 4 | 4 | 0 | 0 | 0 | 6 |

**Supplement 4c: Correlations of summer ski operating days with glaciological and meteorological data: GSA level, summer ski ASTR**

| | Stubai | Kaunertal | Kitzstein-horn | Rettenbach-ferner | Tiefen-bachferner | Pitztal | Mölltal | Dach-stein |
|---|---|---|---|---|---|---|---|---|
| Vernagtferner Temp. Summer (Jun-Sept) | **-0.470*** | -0.197 | -0.369 | **-0.408#** | -0.142 | **-0.402#** | 0.149 | **-0.503*** |
| Hoher Sonnblick Temp. Summer (Jun-Aug) | **-0.439#** | -0.280 | **-0.660**** | **-0.542*** | 0.221 | **-0.438#** | -0.141 | **-0.630**** |
| Hoher Sonnblick Temp. SHY (April-Sept) | **-0.679**** | **-0.455#** | **-0.584*** | **-0.652**** | -0.060 | -0.383 | 0.216 | **-0.729**** |
| Hoher Sonnblick Sunshine duration Summer (Jun-Aug) | -0.129 | -0.005 | **-0.427#** | -0.228 | 0.161 | -0.045 | -0.100 | -0.281 |
| Vernagtferner mean snow depth (May 1) converted to water equivalent mm | 0.036 | 0.004 | -0.253 | -0.189 | 0.257 | -0.189 | 0.062 | -0.242 |
| Hoher Sonnblick mean snow depth (May 1) | -0.013 | -0.125 | -0.166 | -0.197 | 0.100 | 0.204 | 0.239 | -0.083 |
| Hoher Sonnblick mean snow depth ASTR summer | 0.045 | -0.156 | -0.023 | 0.160 | -0.380 | 0.282 | **0.492*** | 0.084 |
| Vernagtferner mass balance | **0.458#** | 0.124 | **0.404#** | **0.464#** | -0.036 | 0.328 | 0.089 | **0.461#** |
| Stubacher Sonnblick-kees mass balance | 0.219 | -0.036 | 0.336 | 0.298 | -0.230 | **0.433#** | 0.217 | 0.258 |
| Vernagtferner ELA | -0.312 | 0.046 | -0.194 | -0.330 | 0.091 | -0.286 | -0.250 | -0.297 |
| Stubacher Sonnblick-kees ELA | **-0.441#** | -0.228 | **-0.455#** | **-0.508*** | 0.108 | **-0.476*** | -0.124 | **-0.482*** |
| Vernagtferner AAR | **0.569*** | 0.329 | 0.341 | **0.501*** | 0.234 | 0.175 | -0.150 | **0.471*** |
| Stubacher Sonnblick-kees AAR | **0.427#** | 0.246 | 0.312 | **0.455#** | -0.054 | 0.311 | 0.056 | 0.374 |
| Nr of sig. variables (max 13) | 7 | 1 | 5 | 7 | 0 | 4 | 1 | 6 |
| Nr of sig. variables SHY+MET+ASTR (max 39) | 18 | 5 | 13 | 19 | 0 | 6 | 2 | 17 |
| Share sig. variables | 46.2% | 12.8% | 33.3% | 48.7% | 0.0% | 15.4% | 5.1% | 43.6% |

Out of nine possibly significant **temperature** variables Dachstein shows the maximum of nine statistically significant negative correlations, Rettenbachferner eight, Stubai and Kitzsteinhorn seven, while Kauner- and Pitztal only have five respectively four and Tiefenbachferner and Mölltal none at all. As to be expected, each significant temperature variable is negatively related to summer ski operation days.

Only ten out of 48 correlation tests with glacier **mass balances** are statistically significant and, as expected, positive. All Stubai and Dachstein operating days correlate significantly with Vernagtferner mass balance; for Rettenbachferner this is the case for SHY and ASTR. Interestingly, five of these ten significant correlations with glacier mass balances occur for summer ski ASTR. The **ELA** is significantly related to the operating days in 13 out of 48 cases, among them twelve for Stubacher Sonnblickkees. Similarly, the **AAR** is significantly related to the operating days in also 13 out of 48 cases, mostly for those GSA with significant correlations with mass balance.

**Snow accumulation** in winter is never significantly related to the summer ski operation days, while the **snow depth** in the **summer season** (Hoher Sonnblick) is only significant twice, both for the neighboring Mölltal GSA.

**Sunshine duration** is only significant for Kitzsteinhorn MET and ASTR summer ski seasons (negative correlation); Kitzsteinhorn is also located relatively near to Hoher Sonnblick observatory, so its values might best reflect the actual weather conditions there.
